# Efficacy of non-instrumental Endodontic treatment in primary teeth: a systematic review of clinical randomized trials

**DOI:** 10.1186/s13643-024-02505-4

**Published:** 2024-04-25

**Authors:** Farah Chouchene, Amira Oueslati, Fatma Masmoudi, Ahlem Baaziz, Fethi Maatouk, Hichem Ghedira

**Affiliations:** Faculty of Dental Medicine of Monastir, University of Monastir. Laboratory of Biological, Clinical and Dento-Facial Approach, University of Monastir, Monastir, Tunisia., 5019 Monastir, Tunisia

**Keywords:** Pulpotomy, Tooth, Deciduous, Anti-bacterial agents, Dental pulp necrosis, Root canal therapy

## Abstract

**Background:**

Endodontic therapy in pediatric dentistry is a challenging procedure, especially for special needs, uncooperative, and very young patients. A new conservative approach which is the non-instrumental endodontic treatment (NIET) has been developed to simplify the management of primary teeth requiring pulpectomy. This review aimed to compare the efficiency of NIET and conventional endodontic treatment in primary teeth.

**Methods:**

Electronic databases including MEDLINE (via PubMed), Cochrane Library (CENTRAL), and Scopus without restrictions on publication year or publication language were searched. Only randomized clinical trials reporting clinical and radiographical outcomes of NIET and conventional pulpectomy on primary teeth were considered eligible.

Two reviewers extracted the data according to the PRISMA statement and assessed the bias risk using the revised Cochrane risk-of-bias tool and a meta-analysis was performed.

**Results:**

From 3322 screened articles, seven articles meeting the inclusion criteria were included. The selected studies included 283 primary molars, of 213 children aged between 3 and 9 years, treated by NIET and conventional pulpectomy, and had follow-up periods ranging from 1 month to tooth exfoliation. Two studies reported good success rates for both the NIET technique and endodontic therapy with no statistically significant difference while three studies showed radiographical significant differences with a low success rate for the NIET technique. Only one study reported better outcomes in the pulpectomy group with statistically significant differences. The quantitative grouping of the included studies showed no significant differences between NIET and conventional endodontic therapy regarding clinical and radiographical success (*p* value > 0.05).

**Conclusion:**

No difference between the NIET technique and the conventional endodontic therapy in primary molars requiring pulpectomy could be confirmed. Results of the present review need to be interpreted with caution since the quality of evidence according to the GRADE was considered as moderate to very low. Therefore, additional clinical trials on the NIET technique are recommended.

## Background

Despite the progress made in the prevention of tooth decay and the widespread natural dentition protection, the early loss of the primary teeth is still a very common problem [1].

Premature loss of primary teeth may cause arch length loss, and mesialization of permanent teeth in addition to ultimately malocclusions [2].

Today, the frequency of tooth decay in children benefiting from decay-prevention programs is lower, but protective dental applications are not fully implemented, and in many regions, deep decay lesions are found in children presenting inadequate oral hygiene education and poor dietary habits [3].

The low thickness of the enamel and dentine structures in the primary teeth, as well as, the coronal, pulpal, and root morphology are responsible for the rapid progression of carious lesions toward the pulp, which often calls for endodontic treatments [4].

To preserve the primary teeth on the arch as long as possible and increase the success of endodontic treatments, several explorations have been undertaken in the last years to find new materials and techniques [5].

Endodontic therapy which is aimed at the elimination of bacteria from the infected root canal is still today a challenging procedure, especially for pediatric patients [3].

In a special category of pediatric patients such as uncooperative and special needs patients, a different approach was indispensable to make the treatment simple and require less time [6].

Due to the complexity of root canals, physiologic root resorption, root curvature in molars, and multiple accessory canals, obtaining a hermetic seal in the apical portion of the primary teeth root can be difficult [7].

Non-instrumental endodontic treatment (NIET) with no mechanical instrumentation are new biological approaches in the treatment of carious lesions with pulpal and with or without periapical involvement using bacteriostatic and bactericides’ agents [8].

Several methods of locally applying antibiotic-based agents for the management of pulp bacterial infections have been well-reviewed [9].

In Latin America, an antibiotic-based paste containing a mixture of chloramphenicol, tetracycline, zinc oxide, and eugenol, named CTZ, has been used for years as a pulpotomy agent in the infected or inflamed pulp, precluding root canal instrumentation [10]. In recent years, the cardiology research unit of the Niigata University, School of Dentistry in Japan, has developed the concept of non-instrumental endodontic treatment named also lesion sterilization and tissue repair (LSTR) therapy [10].

This technique allows disinfection of dentinal, pulpal, and periradicular lesions using a topical application of a mixture of three antibiotics: metronidazole, minocycline, and ciprofloxacin, which are mixed with propylene glycol and polyethylene glycol (MP) as a carrier, the so-called 3Mix-MP [10].

The 3mix-MP can easily distribute through these regions and induce a sterile zone which was expected to promote tissue repair [11].

Hoshino et al. in 1990 used combinations of metronidazole 500 mg, ciprofloxacin 200 mg, and minocycline 100 mg in a 1:1:1 ratio. Takushige et al. in 1998 used the above antibiotics in the ratio 1:3:3 [10].

Despite all the advantages of this method, its use has not been well documented and there is a need to synthesize research evidence.

Thus, the interest in conducting this systematic review which aimed to compare the efficacy of the non-instrumentation endodontic treatment and the conventional endodontic therapy in primary molars requiring pulpectomy by assessing the clinical and radiographical outcomes.

## Material and methods

### Protocol and registration

This systematic review was reported according to the Preferred Reporting Items for systematic reviews and meta‐analysis (PRISMA) guidelines [12].

The protocol was registered in the International Prospective Register of Systematic Reviews database (PROSPERO) under the number CRD42020155273.

### Review question

The present review focused on the following research question: Are non-instrumentation endodontic techniques more effective than conventional pulpectomy techniques in primary teeth?

PICO (Population, Intervention, Control, and outcome) schema for all the included studies to elaborate upon this research question was used to establish the eligibility criteria as follows:

- Population: pediatric patients with teeth requiring pulpectomy.
- Intervention: non-instrumentation endodontic treatment.
- Control: conventional pulpectomy techniques.
- Outcomes: clinical and radiographical success rate.

### Inclusion and exclusion criteria

Randomized controlled trials (RCTs), comparing the clinical and radiographical outcomes of NIET and conventional pulpectomy techniques of primary teeth were included in the present review.

Only studies reporting clinical and radiographical outcomes of non-instrumental pulpectomy and conventional pulpectomy on primary teeth with at least a follow-up of 6 months were considered eligible. The exclusion criteria were as follows: case reports, reviews, cross‐sectional, retrospective, in vitro and animal studies.

All studies investigating pulpotomy in primary teeth, traumatic teeth, or primary teeth without a succedaneums tooth, and pulpectomy in permanent teeth were also excluded in this review.

### Search strategy and data extraction

Search strategies were designed to identify studies reporting clinical and radiographical outcomes of NIET and conventional pulpectomy on primary teeth.

The databases used for research were MEDLINE (via PubMed), The Cochrane Library (CENTRAL), Scopus, and ScienceDirect, without restrictions on publication year or publication language.

The initial search was conducted by the two authors (F.C and A.O) in February 2020 and a subsequent search was performed on July 2023.

A manual search in journals of pediatric dentistry and endodontics related to the topic of interest was performed by the two authors (F.C and A.O) in addition to the electronic database search.

Electronic database searches were supplemented with forwarding citation tracking via Google Scholar and the reference lists of full-text studies were also screened by the two authors (F.C and A.O).

The following search terms and combinations of Medical Subject Heading terms (MeSh) and keywords/text words were used and adapted for each database: (Pulpectomy OR Root Canal Preparation OR Root Canal Therapy OR Root Canal Treatment) AND (LSTR OR NIET OR Non-instrumentation Endodontic Treatment) AND (Tooth, Deciduous).

The set of keywords used during the search is given in Table 1.

**Table 1:** Keywords used to develop the search strategies

Database	Keywords	*N*
PubMed	(("Dental Pulp Necrosis"[Mesh]) AND "Anti-Bacterial Agents"[Mesh]) AND "Tooth, Deciduous"[Mesh] (("Tooth, Deciduous"[Mesh]) AND "Root Canal Therapy"[Mesh]) AND "Anti-Bacterial Agents"[Mesh] (("Pulpectomy"[Mesh]) AND "Anti-Bacterial Agents"[Mesh]) AND "Tooth, Deciduous"[Mesh] (("Anti-Bacterial Agents"[Mesh]) AND "Root Canal Preparation"[Mesh]) AND "Tooth, Deciduous"[Mesh] (("Root Canal Therapy"[Mesh]) OR “Root canal preparation"[Mesh]) AND "Anti-Bacterial Agents"[Mesh] AND "Tooth, Deciduous"[Mesh] (("Metronidazole"[Mesh]) OR "Ciprofloxacin"[Mesh]) AND "Minocycline"[Mesh]) AND "Tinidazole"[Mesh]) AND "Tetracycline"[Mesh]) AND "Ornidazole"[Mesh]) AND "Clindamycin"[Mesh]) OR "Anti-Bacterial Agents"[Mesh] OR “zinc oxide” OR eugenol OR) AND ( "Root Canal Preparation"[Mesh] OR "Root Canal Therapy"[Mesh])) AND "Tooth, Deciduous"[Mesh] ((((((((((Primary[Title/Abstract])) OR (decidious[Title/Abstract])) OR (Children[Title/Abstract])) OR (Pediatric[Title/Abstract])) AND (3Mix[Title/Abstract])) OR (Pulpectomy[Title/Abstract])) OR (Non instrumental[Title/Abstract])	214
Cochrane Library	“#1 Dental pulp necrosis” “#2 Anti-bacterial agents” “#3 Root canal therapy” “#4 Root canal preparation” “#5 Pulpectomy” “#6 Deciduous tooth” “#7- #1 AND #2 AND #3” “#8- #6 AND #3 AND #2” “#9- #5 AND #2 AND #6” “#10- #2 AND #4 AND #6” “#11- #3 OR #3 AND #2 AND #6” “#12 Metronidazole” “#13 Ciprofloxacin” “#14 Minocycline” “#15 Tinidazole” “#16 Tetracycline” “#17 Ornidazole” “#18 Clindamycin” “#19 Agent antimycobacterial” “#20 Antibiotic” “#21 Antibiotic paste” “#22 noninstrumentation endodontic” “#23 lstr” “#24 root canal treatment” “#25- #12 OR #12 OR #13 OR #14 OR #15 OR #16 OR #17 OR #18 OR #19 OR #20 OR #21 AND #5 AND #6” “#26- #5 OR #3 OR #4 OR #22 OR #23 OR #24 AND #6”	2343
Scopus	TITLE-ABS-KEY ("Dental Pulp Necrosis" AND "Anti-Bacterial Agents" AND "Tooth, Deciduous") TITLE-ABS-KEY ("Tooth, Deciduous" AND "Root Canal Therapy" AND "Anti-Bacterial Agents") TITLE-ABS-KEY ("Pulpectomy" AND "Anti-Bacterial Agents" AND "Tooth, Deciduous") TITLE-ABS-KEY ( "Anti-Bacterial Agents" AND "Root Canal Preparation" AND "Tooth, Deciduous") TITLE-ABS-KEY ("Root Canal Therapy" OR “Root canal preparation" AND "Anti-Bacterial Agents" AND "Tooth, Deciduous")TITLE-ABS-KEY( "metronidazole" OR "ciprofloxacin" OR "minocycline" OR "tinidazole" "tetracycline" OR "agents antibacterial" OR "agentsantibacterial" OR "agentsantimycobacterial" OR "antibiotic" OR "antibiotic paste" OR “zinc oxide” OR eugenol ORAND "Pulpectomy" AND "Deciduous teeth") TITLE-ABS-KEY ( "pulpectomy" OR "root canal preparation" OR "canal preparation root" OR "root canal therapy" OR "canal therapies root" OR "root canal procedures" OR "root canal treatment" OR "lstr" OR "noninstrumentation endodontic" AND "Deciduous teeth")	765

Legend: Search strategies used in different databases; PubMed, Cochrane Library, Scopus

The results obtained through the search of the databases, journals, and grey literature (via opengrey.eu) were managed systematically using Mendeley Desktop 1.19.4.

#### Selection of the studies

The titles of all studies were reviewed by two authors (F.C and A.O) independently and the duplicate studies were excluded.

After title selection, the abstracts were reviewed by the two authors (F.C and A.O).

Studies were excluded when no clinical and radiographical outcomes were discussed.

The selected studies were downloaded as full-text papers and then screened by the reviewers.

The references list of the selected articles was also screened for additional data.

The disagreement was settled by a third reviewer (F.M).

#### Data extraction

Using a Microsoft Excel customized data sheet, the two authors (F.C and A.O) independently collected data from the selected studies.

Information and data applicable to the following parameters were extracted from each trial: authors, year of publication, geographic location; study design, demographic details of the participants, sample size, groups according to the type of pulpectomy technique (non-instrumentation or conventional), number of visits, irrigants used, root filling materials used, type of antibiotics mixture (Ratio/placement), type of tooth restoration provided, Follow-up periods and outcomes.

The treatment success was defined based on the accomplishment of specific clinical and radiographical criteria.

There were no restrictions on sample size or the maximum follow-up period.

The clinical criteria were defined as follows: no pain, no swelling, no abscess, and/or decreased in mobility.

The radiographical criteria were considered as a decrease, no changes, or absence of radiolucency when comparing postoperative imaging with X‐rays taken preoperatively.

According to an adaptation of Strindberg’s criteria and following a core set of component outcomes proposed by Smaïl-Faugeron et al. [13] to define failure of a pulp treatment in primary teeth, a treatment was considered a failure if one of the symptoms just described above was reported.

#### Quality assessment

Two authors (F.C and A.O) independently assessed the quality of the methodology and the results outcomes of the included studies using the revised Cochrane risk-of-bias tool for randomized trials (RoB 2.0) [14].

To assess each included trial for risk of bias, five domains were rated: (D1) randomization process; (D2) deviations from intended interventions; (D3) missing outcome data; (D4) measurement of the outcomes; and (D5) selection of the reported results.

The RoB 2.0 was conceived hierarchically and the two authors (F.C and A.O) were asked to answer the signaling questions that provide the basis for domain-level judgements about the risk of bias and evaluate the overall bias of each included study according to the algorithm explained by RoB 2.0 guidance [15].

#### Data synthesis

Direct evidence was computed using a random-effects model meta-analysis and presented as a forest plot with a 95% confidence interval (CI).

Pooled-effect estimates were obtained comparing the overall effective rate of clinical and radiological root healing and this information was reported as the risk ratio (RR).

*P* < 0.5 was considered statistically significant (*Z* test).

Heterogeneity was evaluated using the *I*^*2*^ test; values of *I*^*2*^ higher than 75% led to a moderate heterogeneity of the data in the included studies. The heterogeneity was also evaluated graphically, across the analysis of the overlapping of the confidence intervals in the forest plot.

For the meta-analysis, the software Reviewer Manager 5.4 (Cochrane Collaboration, Copenhagen, Denmark) was used.

## Results

**Fig. 1:**
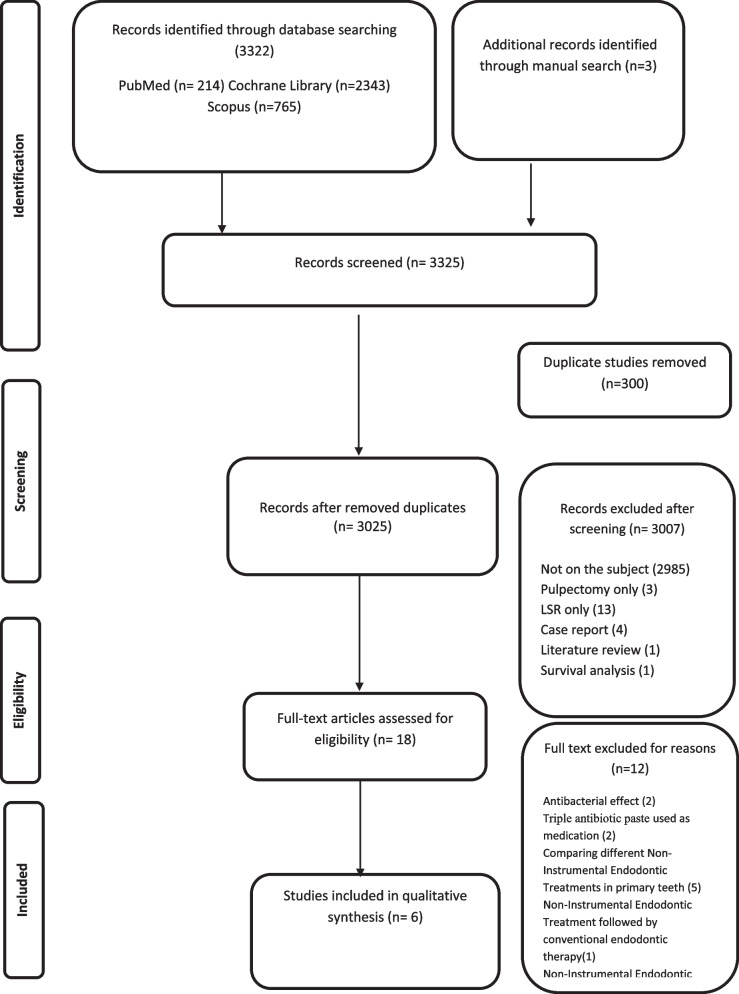
Prisma flow diagram

Details about the process of identification, inclusion, and exclusion of studies are summarized in Fig. 1, Table 2.

**Table 2:** Overview of inclusion and evaluation criteria of the selected studies

**Criterion specification**	**Nakornchai et al. 2010** [16]	**Aagarwal et al. 2011** [17]	**Daher et al. 2015** [18]	**Doneria et al. 2017** [19]	**Divya** **et al. 2019** [20]	**Zacharczuk et al. 2019** [21]
**Clinical inclusion criterion**						
Spontaneous pain	Nm	*	*	*	*	Nm
Tenderness to percussion	Nm	Nm	*	*	Nm	Nm
Signs/symptoms of pulpal pathology (indicating pulpectomy)	Pulpotomized tooth failure	*	*	*	Nm	Nm
**Diagnosis of pulp necrosis**	*	*	*	*	*	*
Abcess, fistula	*	*	*	*	*	Nm
Clinical mobility	*	Nm	*	Nm	*	Nm
**Radiographic inclusion criterion**						
Periapical or bifurcation radiolucency	*	*	*	*	*	Nm
Pathological external root resorption	*	Nm	Nm	Nm	Nm	Nm
Pathological internal root resorption	*	Nm	Nm	*	Nm	Nm
**Clinical evaluation criterion**						
A decrease or an absence of radiolucency	*	*	*	*	*	Nm
**Radiographic inclusion criterion**						
No pain, no swelling, no abscess, no pain on percussion, and/or decreased **mobility**	*	*	*	*	*	*
**Exclusion criteria**						
Non-**restorable teeth, teeth with severe root resorption or severe bone loss, teeth with perforation**	*	*	Nm	*	*	*
Internal resorption	Nm	*	Nm	* Excessive internal resorption	*	*

Legend: Details of inclusion and exclusion criteria of the selected studies

*Nm* not mentioned

^*^Mentioned

All 3322 articles retrieved from the electronic databases were screened by Mendeley Desktop software 1.19.4, which served as a facilitating tool in the organization of the references.

Of these 3322 initial references initially identified, 300 were duplicates and 3 were identified through other sourcing. About 3007 records were excluded by screening for title and abstract, ending with eighteen articles for full-text reading. Twelve records were excluded. Therefore, six studies were included in the present systematic review.

A standardized eligibility drawing sheet was used to register the reasons for exclusion at this stage.

The reasons for the exclusions are described in Fig. 1.

As the data were very heterogeneous, it was difficult to do the meta-analysis. All the studies were subject to qualitative analyses.

### Study characteristics

Table 3 presents the characteristics of the six selected searches for the present systematic review.

**Table 3:** A summary of the descriptive characteristics of the included studies (*N* = 6)

Author, year, country	Study design	Subjects: (participants/age/tooth type)	Restoration	Follow-up (month)		Intervention group		Control group		Conclusions
						Type (ratio/placement)	Success rate	Type	Success rate	
Nakornchai et al Thailand 2010 [16]	RCT	37 children 3–8 years 50 primary molars	GIC Stainless steel crowns (same visit)	6 12		3 Mix-MP: Ciprofloxacin Metronidazole Minocycline 1:1:1/root canal entrance 2.5% NaOCl	Overall Clinical: 6 months: 100% 12 months: 96% Radiographic: 6 months: 84% 12 months: 76%	Vitapex K files 2.5% NaOCl	Clinical: 6 months: 100% 12 months:96% Radiographic: 6 months: 80% 12 months: 56%	Clinical success at 12 months: no statistically significant difference between the two groups (*P* > 0.005). Radiographic success at 12 months: no statistically significant difference between the two groups (*P* > 0.005)
Agarwal et al India 2011 [17]	RCT	34 children 4–9 years 40 primary molars	GIC Stainless steel crowns (after 24 h)	1 3 6 12		3 Mix-MP: Ciprofloxacin Metronidazole Cefixime 1:1:1/root canal entrance Normal saline	Overall: Nm 6 months: 38.89% 12 months: 33.33%	Zinc oxide eugenol cement H files 5% NAOCl	Overall: 78.5% 6 months: 92.86% 12 months: 78.57%	A significant difference in the proportion of failure/success among the groups (*p* = 0.016)
Daher et al Brazil 2015 [18]	RCT	36 children 3–10 (6.2 ± 1.5) years 53 primary molars	GIC + Composite restoration	1 3 6 12, 18, 24/until exfoliation		CTZ paste: Chloramphenicol Tetracycline, Zinc oxide-eugenol 1:1:2/radicular pulp stumps	Overall: 27%	Calcium hydroxide paste (Calcium hydroxide, zinc oxide, propylene glycol) K-files 1% NaOCL	Overall: 68.7%	Low survival rate of NIET in a 2-year follow-up (*P* = 0.02); The radiographic ineffectiveness discourages its use instead of conventional root canal treatment
Doneria et al India 2017 [19]	RCT	43 children 4–8 years 64 primary molars	GIC (same visit) Stainless steel crowns (after 15 days)	6 12		3 Mix-MP: Ornidazole Ciprofloxacin Cefaclor 1:1:1/medication cavity 1% NaOCL	Clinical: 6 months: 95.5% 12 months: 95.5% Radiographic 6 months: 83.5% 12 months: 79.2%	ZnO–OO H Files 1% NaOCL /saline	Clinical: 6 months: 100% 12 months: 100% Radiographic 6 months: 100% 12 months: 100%	Clinical success at 12 months: no statistically significant difference between the two groups (*P* = 0.429). Radiographic success at 12 months: a statistically significant difference between the two groups (*P* = 0.011): Internal resorption was observed in three teeth treated with modified 3 Mix‑MP paste
								Vitapex H Files 1% NaOCL + saline	Clinical: 6 months: 100% 12 months: 100% Radiographic 6 months: 100% 12 months:100%	
Zacharczuk et al Argentina 2019 [21]	RCT	46 children 6.15 ± 1.38 years 6.3 ± 1.49 years 46 primary molars	ZOE Stainless steel (after 7 days)		1 3 6 12 18	3 Mix-MP: Ciprofloxacin Metronidazole Minocycline 1:1:1/Nm 2.5% NaOCL	Clinical: 6 months: 90% 12 months: 83.3% 18 months: 82.3% Overall: 87.5% Radiographic: 6 months: 80% 12 months:77.7% 18 months:76.4% Overall: 82%	Maisto-Capurro paste K Files 1% NaOCL	Clinical 6 months: 89.4% 12 months: 88.8% 18 months: 88.8 Overall: 91.5% Radiographic: 6 months 84.2% 12 months: 83.3% 18 months: 83.3% Overall: 88.3%	No significant clinical or radiographic difference between groups (*P* = 0.48, 031) Both treatments showed similar clinical and radiographic results during the study periods
Divya et al India 2019 [20]	RCT	17 children 4–9 years 30 primary molars	GIC Stainless steel crowns (Same visit)		3 6 12	3 Mix MP: Ciprofloxacin Metronidazole Minocycline 1:3:3:/pulp chamber floor 3% NaOCL	Clinical: 6 months: 100% 12 months: 93% Radiographic: 6 months: 20% 12 months: 60% Overall: 60%	Propolis liquid-mixed Endoflas powder mixture (2:1) K Files 3% NaOCL/Saline	Clinical: 6 months: 100% 12 months: 100% Overall: 100% Radiographic: 6 months: 40% 12 months: 100% Overall: 100%	Propolis liquid mixed Endoflas powder combination showed better results than NIET The difference in the radiograph success rate between the two groups was statistically significant (*p* < 0.05)

*RCT* randomized controlled study, *GIC* glass ionomer cement, *3 Mix-MP* 3 mixture and macrogol-propylene glycol, *ZnO-OO* zinc oxide-ozonated oil, *H-files* Hedstrom file, *K-files* Kerr file, *NIET* non-instrumental endodontic treatment, *Nm* not mentioned, *NaOCL* sodium hypochlorite

The six included searches were published during the last 10 years and were performed in four different countries: India [14, 19, 20], Thailand [15], Argentina [21], and Brazil [18].

The selected studies included 283 primary molars, of 213 children aged between 3 and 9 years, treated by NIET and conventional pulpectomy, and had follow-up periods ranging from 1 month to tooth exfoliation.

The inclusion and evaluation criteria of the selected studies are summarized in Table 2.

All the clinical procedures were performed under rubber dam isolation [14, 15, 18–21].

In each tooth, a cavity was prepared depending upon the extent of the lesion, and caries were removed with no overhanging tooth structure left in to provide good access to the coronal pulp.

Only the pulp chamber was removed and no root canal treatment was performed on the included teeth receiving NIET [14, 15, 18–21].

For teeth receiving conventional endodontic treatment, the radicular pulp was removed using K-files in four studies [15, 18, 20, 21], and H-files in two studies [14, 19].

For NIET technique, the mixture of metronidazole, ciprofloxacin, and minocycline (1:1:1) was used in two studies [15, 21], the mixture of metronidazole, ciprofloxacine, and minocycline (1:3:3) in one study [20], the mixture of metronidazole, ciprofloxacin, and Cefixime (1:1:1) in one study [14], the mixture of ornidazole, ciprofloxacin, and Cefaclor in one study [19], and CTZ paste which is a mixture of chloramphenicol, tetracycline and zinc oxide eugenol (1:1:2) in one study [18].

In all the included studies [15, 17–21] using 3 Mix, carriers: macrogol and propylene glycol were added to the different antibiotics powder mixtures until a consistent non-friable paste was obtained. For conventional endodontic therapy, several canals filling pastes were used: Vitapex, Zinc oxide eugenol cement, Maistro-Cappuro paste, Calcium hydroxide paste, Zinc oxide-ozonated oil (ZnO-OO), Propolis liquid-mixed Endoflas powder [15, 17–21].

Before placement of the 3 Mix-MP in teeth receiving NIET, irrigation was done with 1% sodium hypochlorite [19], 2.5% sodium hypochlorite [15, 21], 3% sodium hypochlorite [20], and normal saline [19].

In conventional endodontic therapy, the following irrigants were used: 5% sodium hypochlorite [14], 3% sodium hypochlorite [20], 2.5% sodium hypochlorite [15], 1% sodium hypochlorite [18, 21], and 1% sodium hypochlorite with normal saline [19].

Final teeth restorations were done using stainless steel crowns in five studies [15, 17, 19–21] and with composite restoration in only one study [18].

Teeth were sealed with glass ionomer cement and then restored with stainless steel crowns in one visit in two studies [15, 20], sealed with glass ionomer cement at the same visit then restored with stainless steel crowns after 24 h in one study [17], sealed with glass ionomer cement at the same visit then restored with stainless steel crowns after 15 days in one study [19], filled with a temporary dressing (zinc oxide eugenol) and then restored with stainless steel crowns after 7 days in one study [21], restored only with composite restoration in one study [18].

### Main outcomes

Three studies showed that the radiographical success rate of conventional pulpectomy was higher than the NIET [14, 18, 19].

No significant difference was found between NIET and conventional pulpectomy technique in the two included studies [20, 21] whereas one study showed a significant difference between both techniques with better results for endodontic therapy [15].

At 6 months follow-up, the clinical success rate of conventional pulpectomy ranged from 89.4 to 100%, while for NIET, the clinical success rate ranged from 38.8 to 100%.

At 12 months follow-up, the clinical success rate of conventional pulpectomy ranged from 89.4 to 100%, and for NIET, it ranged from 33.33 to 96%.

Concerning the radiographical success rate, at 6 months observation period, the radiographical success rate of conventional pulpectomy ranged from 80 to 100% and for NIET technique from 20 to 84%. At 12 months follow-up, the radiographical success rate of conventional pulpectomy ranged from 80 to 100% and for NIET technique from 20 to 84%.

### Risk of bias appraisal

**Fig. 2:**
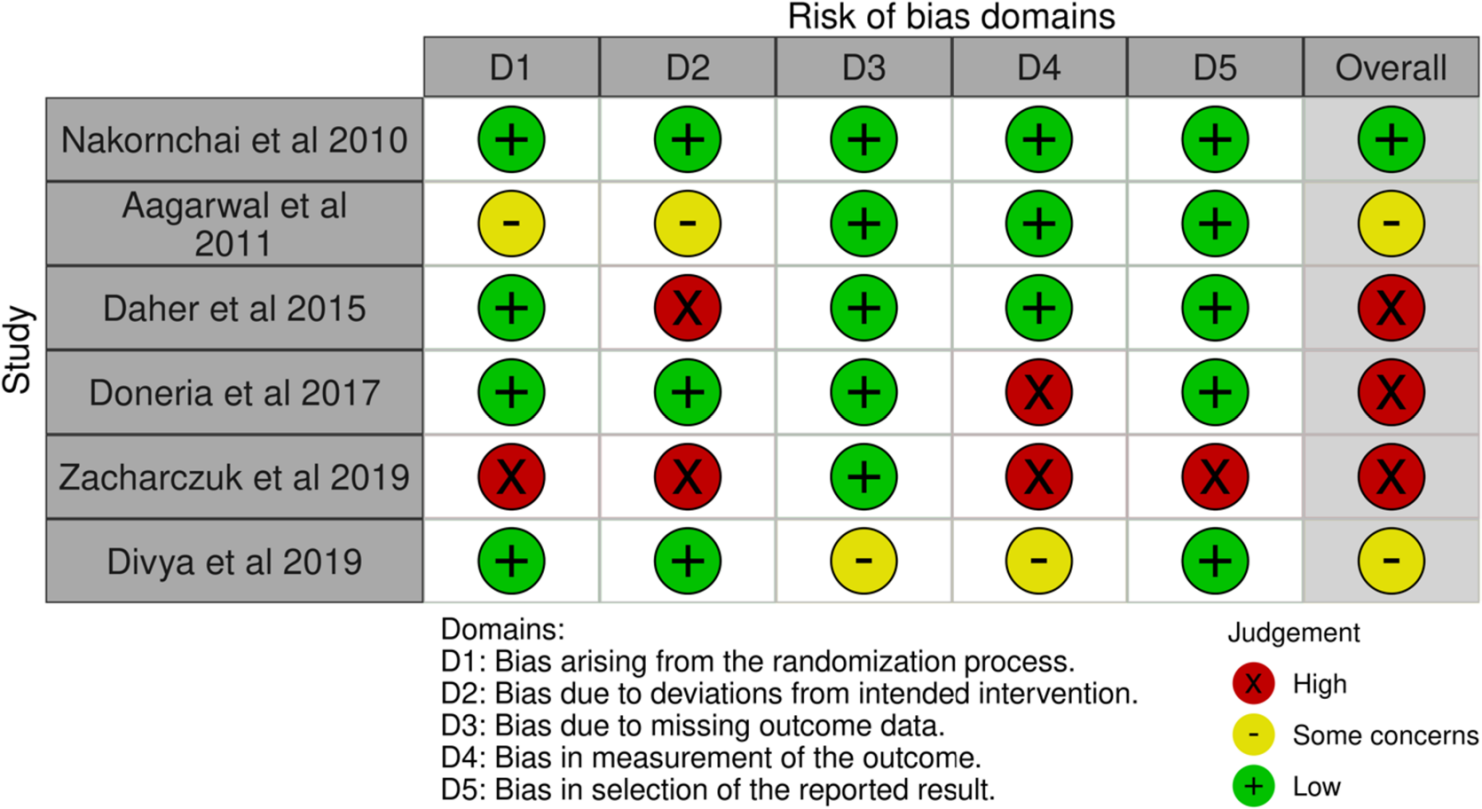
Risk of bias summary

The risk-of-bias assessment summarized in Fig. 2 was generated by the robvis (visualization tool) which is a web application designed for visualizing risk-of-bias assessment [16]. All studies stated acceptable reasons for missing data, with no major missing outcome data.

The overall quality of the randomized clinical trials included in the present review was moderate.

Three studies; Daher et al. 2015 [18], Doneria et al. 2015 [19], and Zacharczuk et al. 2019 [21], were considered as “high risk of bias”.

In these studies, a lack of information about the random sequence generation and allocation was reported and no blinded clinical evaluations were noted in these studies.

The study of Agrawal et al. 2011 [17], and the study of Divyia et al. 2019 [20] were classified as having “unclear risk of bias” due to some concerns reported in the randomization process and the outcomes measurement.

Only one study, Nakornchai et al. 2010 [16], was classified as having a low risk of bias.

### Meta-analysis

**Fig. 3:**
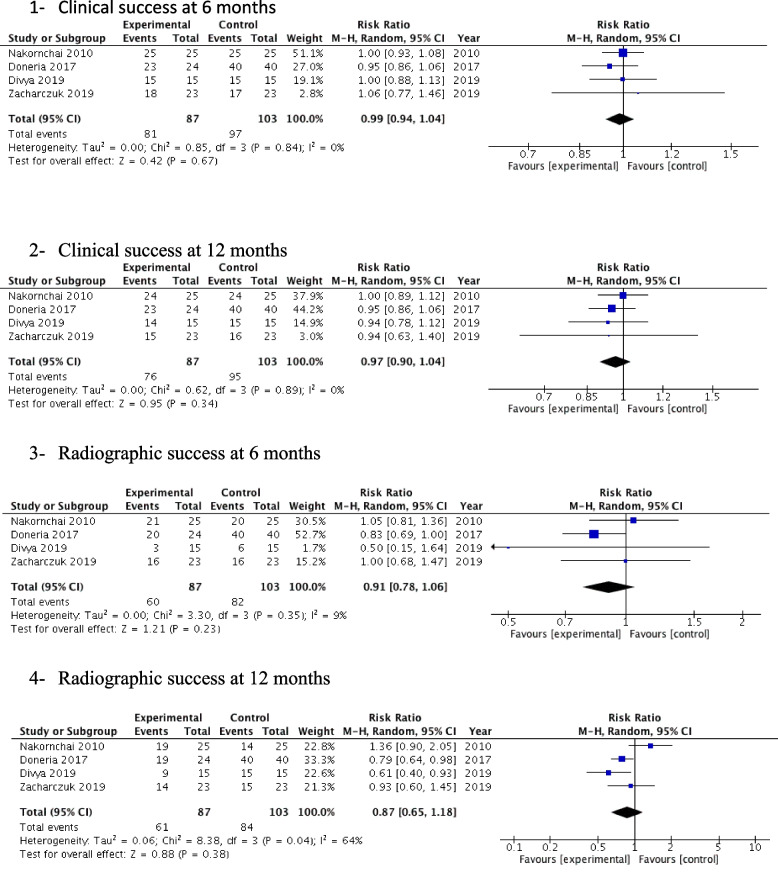
Forest plot of treatments clinical and radiographic parameters at 6 and 12 months

Of the 6 included studies in the present systematic review, only 4 were included in the meta-analysis (Fig. 3).

At 6-month follow-up, there was no significant difference in the clinical and radiographical success rates between the non-instrumentation endodontic treatment and the conventional endodontic therapy in primary molars.

The clinical results at 6 months showed; a relative risk (RR) = 0.99, with a 95% CI 0.94, 1.04, and a *P* value = 0.64 with *I*^2^ = 0%.

The radiographical results at 6 months showed; a relative risk (RR) = 0.91, with a 95% CI 0.78, 1.06 and a *P* value = 0.23 with *I*^2^ = 9%.

At 12-month follow-up, no significant difference in the clinical and radiographical success rates between the non-instrumentation endodontic treatment and the conventional endodontic therapy were reported.

The clinical results at 12 months showed; a relative risk (RR) = 0.97, with a 95% CI 0.90, 1.04, and a *P* value = 0.34 with *I*^2^ = 0%.

The radiographical results at 12 months showed; a relative risk (RR) = 0.87, with a 95% CI 0.65, 1.18 and a *P* value = 0.38 with *I*^2^ = 64%.

A meta-regression was not performed as it is not recommended when the number of the included studies is small [22].

## Discussion

This systematic review aimed to compare the efficacy of the Non-Instrumentation Endodontic Treatment and the conventional endodontic therapy in non-vital primary molars requiring pulpectomy.

According to the present review, no difference between the NIET and the conventional endodontic therapy in primary molars requiring pulpectomy could be confirmed.

The NIET technique was proposed as an alternative to endodontic therapy especially for uncooperative children and low-resource areas [10]. This therapy was described as a simple not time-consuming and one-visit technique [11]. It requires no mechanical instrumentation because the antimicrobial capacity of the antibiotic paste sterilizes the area promoting lesion repair and preserving the primary tooth until normal exfoliation time [23].

In this review, Doneria et al. and Divya et al. [19, 20] reported high clinical success rates at 12 months follow-up in NIET and conventional endodontic therapy.

In several included studies, clinical symptom remission was found, and among the selected groups when comparing the pre-and post-operative spontaneous pain and tenderness to percussion a statistically significant difference was observed [17–19].

Concerning radiographical success, the radiographical success rate in NIET groups differs from one study to another [19, 20]. Nakornchai et al. [16] reported no significant differences between the NIET group and the control group with better radiographical outcomes in the NIET group. This result was in contrast with Doneria et al. [19].

In the Nakornchai et al. study [16], non-vital primary teeth with ½ root resorption were selected which may explain the reported results.

Therefore, the degree of preoperative root resorption should be considered as an important parameter influencing the choice of the most convenient obturation technique [15].

In the present review, two studies reported a significant difference with a low success rate for NIET comparing to conventional endodontic treatment [17, 18].

The low overall success rate of NIET technique in Agarwal et al. study [17] was attributed essentially to the low radiographical success where radicular radiolucency and internal resorption were reported at 12 months follow-up.

Daher et al. [18] reported a lower success rate with CTZ paste. These results were due to the low radiographical success and the initial challenging diagnosis [18].

A significantly low survival rate was found for CTZ paste in the necrotic pulp at 26 months and better results were found in vital teeth with irreversible pulpitis [18].

At 12 months follow-up, most of the studies reported a higher radiographical failure rate in the NIET group than the endodontic treatment group.

In the present review, the follow-up period of the included studies was inferior to 26 months [17–21]. Few articles in the literature have studied NIET for a long period [24].

These studies have shown that the efficiency of the NIET technique decreases over time [21, 24].

Results in Grewal et al. [24] study demonstrated that at 12 months follow-up, the 3 Mix paste presented better radiographical and clinical outcomes compared to calcium hydroxide and iodoform used in the conventional endodontic treatment. However, radiographically significant pathological changes were observed at 36 months in NIET-treated teeth which suggests that NIET cannot be advocated as a long-term alternative to the conventional endodontic treatment in primary teeth [24].

Root resorption and strategic tooth position in the arch should be considered before NIET [25].

According to the present review, NIET was recommended when a tooth should be maintained for less than 12 months and exhibits root resorption [17].

The evidence suggests that the NIET and the use of the 3 Mix antibiotics compared to the conventional materials and techniques can help to reduce the inflammation and bacterial contamination and can be considered as an efficient treatment option for non-vital primary teeth.

Regarding antibiotic mixtures, several studies have compared the efficacy of 3 Mix-MP with other mixtures of antibiotics in the endodontic treatment of primary teeth with necrotic pulp [24, 26, 27].

The bacterial composition of the infected root canal is complex, a single antibacterial drug may not be effective for this reason metronidazole was selected as the first choice among antibacterial drugs [18].

Some bacteria were resistant and it was necessary to combine it with other drugs, hence, ciprofloxacin and minocycline were added [28].

Pinky et al. [29] compared two different combinations and no statistically significant difference was found between the use of metronidazole, ciprofloxacin, and minocycline mixture in the first group and ornidazole instead of metronidazole in the second group.

Nanda et al. [8] showed the same results with no statistically significant difference between 3 Mix (ciprofloxacin, metronidazole, minocycline) and other mixes (ciprofloxacin, ornidazole, minocycline). However, a higher radiographical success rate was reported with the modified combination at 12 months for both studies. This could be explained by the presence of ornidazole which has a longer duration of action, slower metabolism, and better efficacy than metronidazole [8].

Jaya et al. [27] showed no differences between the efficacy of metronidazole and trinidazole in combination with ciprofloxacin and minocycline. However, Tinidazole appears to have several advantages over metronidazole including greater in vitro potency against both sensitive and resistant strains of obligate anaerobes, more prolonged duration of action, and improved patient tolerability [27]. This alternative drug combination may be used based on the spectrum of antibacterial activity and availability [27].

Raslan et al. [30] used clindamycin instead of minocycline and reported no statistical difference between 3 Mix-MP and mix-MP-R. Primary teeth with necrotic pulp can be treated with both combinations regardless of the degree of root resorption Primary teeth with necrotic pulp can be treated with both combinations irrespective of the degree of root resorption [30].

The 3 Mix and the Modified 3 Mix drugs were both mentioned in the included studies Primary teeth with necrotic pulp can be treated with both combinations irrespective of the degree of root resorption Primary teeth with necrotic pulp can be treated with both combinations irrespective of the degree of root resorption [15, 17–21].

The present review showed that different combinations could be used and that the metronidazole could be replaced by the tinidazole or the ornidazole and the clindamycin instead of the minocycline [15, 17–21].

The Cardiology Research Unit of Niigata University reported a 1:1:1 ratio of 3 Mix, however, good results were found also with a 1:3:3 ratio in different studies Primary teeth with necrotic pulp can be treated with both combinations irrespective of the degree of root resorption [24].

Although the present review included several studies using different combinations of antibiotics, none of them has reported allergic reactions however the use of antibiotics risks increasing resistant strains [15, 17–21].

Due to the systematic differences within the studies analyzed in the present review, a moderate level of heterogeneity was reported.

Concerning differences in the methods and sample criterion, all included articles studied non-vital primary teeth with periapical lesions while three articles included vital teeth requiring pulpectomy [15, 17–21]. All articles excluded non-restorable teeth with excessive root resorption (more than 2/3 of the root length) or excessive bone loss or perforation [20, 21].

In this review, a lack of information about the random sequence generation and the allocation was noted. In most of the included studies, patients were not blinded or it was not clear. Also, some concerns were reported in the randomization process and the outcomes measurement.

The limited number of studies included in the present meta-analyses, and the limited number of published studies comparing the non-instrumentation endodontic treatment and the conventional endodontic therapy in primary molars requiring pulpectomy can be considered also as a main limitation.

Randomized clinical trials with larger samples, longer follow-up periods, and high-quality study designs to further strengthen the efficacy of NIET are needed.

## Conclusion

NIET can be considered effective on vital pulp with irreversible pulpitis and necrotic teeth without or with periapical lesions (root resorption not exceeding half of the root length) requiring short-term space management. It can be also useful for non-cooperative patients and patients with special needs. In this technique, different antibiotic combinations could be used to sterilize necrotic primary teeth with periapical lesions showing poor prognosis for conventional endodontic treatment.

There is limited evidence on NIET using different antibiotic combinations and results of the present review need to be interpreted with caution. However, more studies should be conducted with larger samples longer follow-up periods, and high-quality study designs to further strengthen the efficacy of NIET.
